# History of a Case of Epilepsy, Cured by Applying a Caustic to the Nerve Which Accompanies the Vena Saphena

**Published:** 1803-07-01

**Authors:** M. Pontier


HISTORY *of a Case of* EPILEPSY, *cured by applying a Caustic to the Nerve which accompanies the Vena Saphena; by M. Pontier. Communicated by our Correspondent at Paris.*

THE author states, that a young man, twenty-five years of age, of a plethoric habit, came into the hospital to be treated for an epilepsy, the fits of which returned three times a day regularly at six and nine in the morning and at two in the afternoon. After the unsuccessful trials of opium, bark, camphire, valerian and moxas, applied to the head, the author recollected to have read in the *Recueil Periodique* of the Medical Society, an observation of an epilepsy cured by the extirpation of a small tumor from the thumb of the right hand. Struck by this fact, and after close enquiry into the history of the complaint, he learned that the patient had been bled in the right foot, some months before, on account of the epilepsy, which had attacked him two years back; that some time after he had been bled in the left, and in consequence of which the duration of the fits became much longer. The author suspected the case to be sympathetic, and to have proceeded from the imperfect section of the nerve accompanying the vena saphena. The incision of the vein was made transversly, and to the touch a small hardness was perceivable of the size of a barley-corn, situated on the outer side of the vein; for eight months the patient had not experienced a particular sensation of cold, which ran up the right leg, and which, anterior to this period, had always troubled him. This circumstance was of importance to the author, who, an instant previous to the usual fit at two o'clock, applied a ligature above the right knee; the fit continued but five minutes instead of twenty-five, the usual period. The same consequences followed the application at nine o'clock The following day the fit was suspended by the same means; and when the tourniquet was used, and the bolster made to press on the course of the nerve in question, the fit was found to continue but a minute and a half. The author observed, that on relaxing the tourniquet at the conclusion of the fit, a general trembling came on, which was suspended by tightening anew the instrument. On applying the ligature above the ankles the fits were in like manner suspended. As yet no pressure was made on the extremity; but the author, in order to assure himself that it was in part the cause, placed a ligature on each leg

E 2 some some time before the period of accession; no fit took place. The patient appearing to have some disposition to sleep, M. Pontier endeavoured to divert him by various means; a disposition to laugh, as if intoxicated, slight tremors of the head, a difficulty in articulating certain words, were appearances which marked the period of the usual attack; but the patient never lost the use of his reason and senses. The same experiments were repeated with the same results the two following days. In the evening, caustic alkali was applied to the cicatrices, with a view to divide completely the nerves; the caustic had produced its effect before the period of the next fit, which did not take place, notwithstanding that no pressure whatever was applied, and from this moment all symptoms disappeared. During the sloughing of the escar, the patient was ordered to take two drachms of valerian and an equal quantity of bark. This case is concluded by the following reflections of one of the editors of the *Recueil Periodique*. Although wounds of nerves be a very frequent cause of epilepsy, yet in the case in question it would not appear to be owing to the wound inflicted in bleeding, since the disease existed some years before. It appears only that the complaint became aggravated after bleeding. We should be desirous to know if the small tumour, marked in the history of the case, existed before the operation; and whether it can be considered a sufficient cause of epilepsy? 2dly, Whether or not the same mode of treatment was not equally indicated before blood-letting? We find, in the annals of the science, a number of cases of epilepsy, where the fits were suspended by the application of ligatures above the place where the aura epileptica was perceived, and where there was no reason for attributing the disease to the imperfect division, or to a wound of a nerve. It has been found too that this application of ligatures persevered in, has sometimes cured the disease. The application of cauteries to different parts of nerves has also produced a cure. For these reasons, would it not seem more correct to give greater extent to the mode of cure than the author has done, and not confine it exclusively to cases where the disease may be supposed to arise from a partial division of a nerve?

HISTORICAL

