# Errata

**Published:** 1803-07-01

**Authors:** 


Vol. x. Page 52, running Title, for *Apoplexy*, read *Epilepsy*.

